# SLC39A6: a potential target for diagnosis and therapy of esophageal carcinoma

**DOI:** 10.1186/s12967-015-0681-z

**Published:** 2015-10-06

**Authors:** Xiao-Bin Cui, Yao-yuan Shen, Ting-ting Jin, Su Li, Ting-ting Li, Shu-mao Zhang, Hao Peng, Chun-xia Liu, Shu-gang Li, Lan Yang, Na Li, Jian-ming Hu, Jin-Fang Jiang, Man Li, Wei-hua Liang, Yong Li, Yu-tao Wei, Zhen-zhu Sun, Chuan-yue Wu, Yun-Zhao Chen, Feng Li

**Affiliations:** Department of Pathology and Key Laboratory for Xinjiang Endemic and Ethnic Diseases, Shihezi University School of Medicine, North 4th Road, 832002 Shihezi, China; Department of Oncology, Tongji Hospital, Huazhong University of Science and Technology, 430030 Wuhan, China; Department of Pathology, People Hospital of Xinjiang Uygur Autonomous Region, Ürümqi, China; Department of Oncology, The First Affiliated Hospital, Shihezi University School of Medicine, 832002 Shihezi, China; Department of CT and MRI, The First Affiliated Hospital, Shihezi University School of Medicine, 832002 Shihezi, China; Department of Thoracic and Cardiovascular Surgery, The First Affiliated Hospital, Shihezi University School of Medicine, 832002 Shihezi, China; Department of Pathology, University of Pittsburgh, Pittsburgh, PA 15261 USA

**Keywords:** SLC39A6, ESCC, Precursor lesions, Prognosis

## Abstract

**Background:**

Esophageal squamous cell carcinoma (ESCC) is a highly lethal cancer, and its underlying molecular mechanisms are poorly understood. Recent large-scale genome-wide association studies in Chinese Han populations have identified an ESCC susceptibility locus within the SLC39A6 gene. Here, we sought to explore the expression and biological function of SLC39A6 in ESCC.

**Methods:**

Multiethnic validation of SLC39A6 protein expression was performed in different cohorts of patients from Chinese Han and Kazakh populations in the Xinjiang region by immunohistochemistry. The associations among SLC39A6 expression, clinicopathological parameters, and prognosis outcomes of ESCC were analyzed. And the effects of SLC39A6 silencing by siRNA on cell proliferation, apoptosis, and invasiveness, as well as the proteins involved in epithelial-to-mesenchymal transition (EMT) of esophageal cancer cells, were studied.

**Results:**

SLC39A6 protein expression increased progressively from normal esophageal epithelium (NEE) to low-grade intraepithelial neoplasia to ESCC, and finally reached the highest in high-grade intraepithelial neoplasia from Han ethnic. Similarly, SLC39A6 protein was significantly overexpressed in Kazakh ethnic ESCC compared with that in NEE. Increased expression of SLC39A6 was found to be closely correlated with histological grade and early Tumor-Node-Metastasis stage I/II. High tumorous SLC39A6 expression was significantly correlated with shorter overall survival (OS). Cox regression analysis confirmed that SLC39A6 expression was an independent prognostic factor for poor OS in ESCC. Experimentally, the suppression of SLC39A6 expression promoted ESCC cell apoptosis but abrogated proliferation and invasion, and induced an EMT phenotype that included enhanced expression of E-cadherin, loss of vimentin, and morphological changes in ESCC cells in vitro.

**Conclusions:**

Combined, our findings highlight a tumor-promoting role for SLC39A6 in ESCC, suggesting that SLC39A6 could serve as an early detector of high-risk subjects and prognostic biomarker. The targeting of SLC39A6 might be a potential therapeutic strategy for blocking ESCC.

**Electronic supplementary material:**

The online version of this article (doi:10.1186/s12967-015-0681-z) contains supplementary material, which is available to authorized users.

## Background

Esophageal squamous cell carcinoma (ESCC), a major histological type of esophageal cancer in East Asian countries, is one of the most lethal cancers in the world [1–3]. ESCC ranks the sixth in mortality and the seventh in incidence in China with great variations in geography, ethnicity, and socio cultures [4]. Despite the development of multimodal therapies, including surgery, chemotherapy, and chemoradiotherapy, the prognosis of patients remains poor. The long-term outcome of this cancer is still dismal, with 5-year survival rates of around 30 % [5, 6]. In contrast, the 5-year survival rate of early esophageal cancer is higher than 90 % [7]. Although a variety of molecular alterations have been identified over the last two decades [8, 9], sensitive and specific biomarkers for early diagnosis and accurate indicators for ESCC prognosis are currently unavailable. It is imperative, therefore, to identify novel biomarkers for early detection and therapeutic targets if long-term survival of ESCC is to be improved.

A large-scale genome-wide association study (GWAS) of a Chinese Han populations recently identified a new ESCC susceptibility locus at chromosome 18q12.2, tagged by a non-synonymous SNP of rs7242481, which is located in the 5′UTR in SLC39A6 [10]. SLC39A6, also known as LIV-1, belongs to a new subfamily of Zrt, Irt-like protein zinc transporters (LZTs) [11]. It is involved in maintaining the intracellular homeostasis of zinc, an ion that is essential in the control of cellular growth and differentiation [12]. SLC39A6 plays a critical role in maintaining zinc homeostasis, and was originally identified as an estrogen-induced gene in a breast cancer cell line [13]. Generally, elevated SLC39A6 expression is reportedly related to cancer progression in other various types of cancer, including breast [13, 14], prostate [15], pancreatic [16], cervical [17] and liver cancers [18]. Conversely, controversial data on the SLC39A6 expression have also been reported in many cancers, such as breast cancer. The reports from the Grattan et al. found that SLC39A6 expression is positively associated with metastasis to regional lymph nodes [13], whereas Kasper et al. reported that SLC39A6 expression is associated with a positive outcome in breast cancer patients [19]. The findings suggested that the correlation of SLC39A6 and prognosis might vary in similar malignancies because of population heterogeneity, so whether it is elevated or suppressed in tumors is dependent on the types and contexts of cancers.

In the case of ESCC, SLC39A6 has received relatively little attention. Evidence for its potential utility as a target gene for prognosis and treatment of esophageal cancer is lacking. Although only one large-scale GWAS reported SLC39A6 as the susceptibility gene of ESCC and the expression level in a Chinese Han population [10], no other report has discussed the expression of SLC39A6 in precursor lesions and other ethnic patients with esophageal carcinoma. Therefore, more studies are necessary to characterize the expression of SLC39A6 during the multi-stage development of esophageal cancer, and determine its prognostic value. In addition, most current studies demonstrated that SLC39A6, as an obligatory co-factor, has a crucial role in regulating epithelial–mesenchymal transition (EMT) in pancreatic [16] and prostate cancers [15], causing cancer cell migration. However, the roles of SLC39A6 in ESCC have not yet been characterized.

In this study, we sought to investigate the expression pattern of SLC39A6 in ESCC patients of two different ethnicities and its precursor lesions tissues, and characterize the underlying biological function of SLC39A6 in ESCC cells. We not only discovered that SLC39A6 could serve as an early detector of high-risk subjects and prognostic biomarker, but also discovered the tumor-promoting role of SLC39A6 in regulating ESCC progression and metastasis via inducing an EMT phenotype. The targeting of SLC39A6 might be a potential therapeutic strategy for blocking ESCC in patients from Han and Kazakh ethnic groups.

## Methods

### Patients and tissue specimens

Tissue microarrays (TMAs) were used for immunostaining of SLC39A6 in two independent cohorts of ESCC. One cohort comprised 142 Han ethnic patients with ESCC collected between 1997 and 2013 from the First University Hospital, Shihezi University School of Medicine. The other cohort comprised 86 Kazakh ethnic patients with ESCC collected between 1984 and 2011 from the First University Hospital, Shihezi University School of Medicine, Xinjiang Yili Prefecture Friendship Hospital and People’s Hospital of Xinjiang Uyghur Autonomous Region. No restrictions were placed in terms of age, sex, or disease stage. None of the patients received prior surgery other than diagnostic biopsies, chemotherapy, or radiation therapy. Clinical data were collected on clinicopathologic variables, such as tumor differentiation and lymph node metastasis. All cases with pathologic diagnoses for tumor-node-metastasis (TNM) stages were evaluated according to Cancer Stage Manual, 7th Edition, issued in 2009 by the American Joint Committee on Cancer (AJCC/UICC). Clinical characteristics of patients from both validation centers are listed in Table 1. As expected, most patients who underwent surgery in both centers were men. However, some notable differences in clinical characteristics were observed between the two centers. Patients from the Han ethnic group were slightly older than those in the Kazakh ethnic group (age 58.0 vs. 63.5 years; P < 0.001). More patients in the Kazakh ethnic cohort had lymph node metastasis compared with patients in the Han ethnic group (56.98 vs. 34.51 %; P < 0.001) (Table 1).

**Table 1:** Demographic and clinical characteristics of patients in validation cohorts

Characteristic	Han ethnic		Kazakh ethnic		P value
	(N = 142)		(N = 86)		
	No.	%	No.	%	
Age at surgery, years					<0.001
Median	63		58		
Range	36–81		34–73		
Sex					0.028
Male	107	75.35	53	61.63	
Female	35	24.65	33	38.37	
Differentiation					0.316
Well	39	27.46	21	24.42	
Moderate	74	52.11	53	61.63	
Poor	29	20.42	12	13.95	
Lymph node metastasis					0.001
No	93	65.49	37	43.02	
Yes	49	34.51	49	56.98	
TNM					0.485
I + II	94	66.2	53	61.63	
III + IV	48	33.8	33	38.37	

P < 0.05 indicates a significant association among the variables

A total of 142 Han ethnic cancer tissues combined with its 133 adjacent mucosa and 132 distant normal mucosa and 86 Kazakh ethnic cancer tissues with its 41 distant normal mucosa were removed immediately after surgery, fixed in 10 % formalin, and embedded in paraffin. Approximately 133 precursor lesions in the adjacent mucosa were selected and classified as 81 low-grade intraepithelial neoplasia (LGIN) and 52 high-grade intraepithelial neoplasia (HGIN) (Additional file 1: Figure S1). All the cases were diagnosed by two pathologists. Clinical and pathological data of all patients were obtained from medical records. Follow-ups were conducted on 75 Han patients, with a follow-up deadline of 10 July 2014. The clinical-pathological characteristics of 75 esophageal cancer patients with follow-up information were presented in the Additional file 2: Table S1.

### SLC39A6 expression detected by immunohistochemistry (IHC) using TMAs

Paraffin-embedded materials were sampled from 228 (142 Han, 86 Kazakh) formalin-fixed esophageal cancer tissues, 133 precursor lesion tissues, and 173 (132 Han, 41 Kazakh) normal tissue samples with 0.6 mm-diameter tissue cores using a tissue arrayer (ALPHELYS, Plaisir, France). Slides were stained according to the manufacturers’ protocols for SLC39A6 (14236-1-AP: Proteintech Group Inc., Chicago, USA) which was a rabbit polyclonal anti-SLC39A6/LIV1 antibody and was consistent with the previously described report [10]. In brief, paraffin-embedded 4 µm sections were baked at 65 °C for 60 min and then rehydrated using graded alcohols, as previously described. Each 4 μm tissue section was deparaffinized and rehydrated. The sections were autoclaved in 1 mM ethylenediaminetetraacetic acid buffer (pH 9.0) at 130 °C for 10 min for anti-SLC39A6, cooled to 30 °C for 40 min, and incubated with fresh 3 % H_2_O_2_ in methanol for 10 min at room temperature. Tissue sections were then incubated at 4 °C overnight with anti-SLC39A6 rabbit polyclonal antibody at a dilution of 1:2400 in PBS containing 1 % bovine serum albumin, washed in PBS, and incubated with secondary antibody for 30 min at 37 °C. Subsequently, 3,3-diaminobenzidine was employed to visualize SLC39A6 antibody binding, and the tissue sections were counterstained with hematoxylin. In addition, negative controls were performed using PBS instead of the SLC39A6 antibody.

### Semi-quantitative assessment and scoring

The expression of SLC39A6 was scored semi-quantitatively according to the percentage of positive cells and cytoplasmic/nuclear staining intensity. The percentage of positively stained cells was as follows: 0 (<5 % positive cells), 1 (6–25 % positive cells), 2 (26–50 % positive cells), 3 (51–75 % positive cells), or 4 (>75 % positive cells). The cytoplasmic/nuclear staining intensity was categorized as follows: 0 score, negative; 1 score, buff; 2 score, yellow; and 3 score, brown. The percentage of positive epithelial cells and staining intensities were then multiplied to obtain the immunoreactivity score (IS) for each case. For example, if the staining intensity was brown (3) and the percentage of positive cells was greater than 45 % (2), then the IS would be 3 × 2 = 6. Two pathologists independently reviewed five random fields from each sample slide. Cases with discrepant scores were reviewed using a 10-headed microscope and re-assigned a consensus score. Thus, the IS range was from 0 to 12. Optimal cut-off values for this assessment system were identified as follows: high expression of SLC39A6 was defined as an expression index score of 5, and low expression of SLC39A6 was defined as an expression index score of < 5. These cases were divided into two groups based on their IS of SLC39A6 staining. Cases with a score of ≥5 were categorized as the high expression group, and cases with a score of <5 were categorized as the low expression group.

### Cell lines and culture conditions

Four esophageal cancer cell lines (Eca109, EC9706, TE-1, and KYSE-150) and a normal esophageal epithelium (NEE) cell line (HEEC) were purchased from the Institute of Biochemistry and Cell Biology of the Chinese Academy of Sciences (Shanghai, China). Cells were cultured in RPMI 1640 or DMEM (GIBCO-BRL) supplemented with 10 % fetal bovine serum, 100 U/mL penicillin, and 100 mg/mL streptomycin in humidified air at 37 °C with 5 % CO_2_.

### Small interfering RNA (siRNA)-based knockdown

Oligonucleotide siRNA duplexes were synthesized by Invitrogen (Carlsbad, USA). The following siRNA sequence for SLC39A6 was used: 5′-UUC CAU UGC UGG UUC UUC AUG GCU A-3′. A non-target scrambled siRNA was used as the negative control: 5′-UUC UCC GAA CGU GUC ACG UTT-A-3′. The cells were transfected with 50 nM siRNA targeting SLC39A6 or RNAi negative control duplexes using HiPerFect transfection reagents (Qiagen, Hilden, Germany) in serum-free conditions according to the Quick-StartProtocol.

### Western blot analysis

Western blot analyses were performed on cell lysates prepared from Eca109 and EC9706 cell lines as described previously. Transfected cells were lysed in RIPA lysis buffer (Solarbio). Cell protein lysates were separated by 10 % SDS-polyacrylamide gel electrophoresis. Proteins were transferred to PVDF membranes (Immobilon 0.45 μm, Millipore, USA), and immersed in a blocking solution containing 5 % non-fat milk and 0.1 % Tween-20 for 1 h. After blocking, membranes were incubated with primary antibodies overnight at 4 °C and then with secondary antibodies for 2 h at room temperature. After washing, the resulting bands were visualized using the standard ECL procedure, quantified by densitometry, and normalized to the corresponding β-actin bands. The following antibodies were also used: anti-SLC39A6 (14236-1-AP: Proteintech Group Inc., Chicago, USA), anti-β-actin (sc-47778, Santa Cruz, Santa Cruz, CA, USA). Antibodies against E-cadherin, Vimentin were purchased from Santa Cruz Biotechnology, Inc. (Santa Cruz, CA, USA).

### In vitro cell growth assay

Cell growth was measured by 3-(4,5-dimethylthiazol-2-yl)-2,5-diphenyl-tetrazolium bromide (MTT) assay. Cells (4 × 10^3^) were seeded at each well of 96-well flat-bottom plates (NUNC). After culturing for 24, 48, 72, 96, and 120 h, cells were stained with 20 μL of sterile MTT dye (5 mg/mL, Solarbio) for 4 h at 37 °C. The culture medium was removed, and 150 μL of DMSO was added. The 96-well plates were shaken until the formazan crystals dissolved completely. The absorbance value was measured on a microplate reader (Bio-Rad) at 490 nm.

### Cell apoptosis assay

Cell apoptosis was analyzed by flow cytometry. Cells were cultured in 24-well plates. At 48 h after transfection, cells were removed from the plate using a trypsin digestion solution, collected, and resuspended in 500 µL of 1 × binding buffer. After the addition of 5 µL of Annexin V-FITC to each well, cells were incubated in the dark for 5 min. An aliquot of 10 µL of PI was added to each well, followed by additional incubation in the dark for 5 min. Finally, flow cytometry was performed.

### Colony formation assay

Cells (3 × 10^3^) were plated in six-well plates for 14 days at 37 °C. The cells were washed with 1 mL of PBS, fixed with 4 % paraformaldehyde for 15 min, stained with 0.1 % crystal violet for 20 min, and finally washed three times with 1 mL of water. The number of colonies was manually counted.

### Matrigel invasion assay

The Matrigel invasion membrane (BD BioCoat Matrigel Invasion Chamber; BD Biosciences) was rehydrated. Cells were added to a Matrigel invasion chamber in triplicate and then incubated for 8 h. After noninvading cells were removed, the membranes containing the invading cells were stained with DAPI.

### Statistical analysis

All statistical analyses were performed using SPSS (IBM Corp., Armonk, NY, USA) version 17.0. To compare differences in demographic and clinical factors between the two validation cohorts, t test was used for continuous variables, and χ^2^ test and Fisher’s exact test were used for categorical variables. The Chi square test or Fisher’s exact test were used to evaluate the associations between SLC39A6 expression and clinicopathological features. McNemar test was used to assess the difference in the proportion of samples with high level of the SLC39A6 protein between the ESCC and their corresponding LGIN and HGIN tissues in Chinese Han population. Receiver operating characteristic (ROC) curves analysis and the area under the curve (AUC) were used to evaluate the specificity and sensitivity of ESCC and esophageal squamous intraepithelial neoplasia (ESIN). Correlations between prognostic outcomes and SLC39A6 expression were investigated using Kaplan–Meier analysis and the Cox proportional hazards model. The significance of prognostic factors on survival was studied by Cox regression model. Data points are reported as experimental average, and error bars represent SD. For the variables, a P value of less than 0.05 was considered statistically significant.

## Results

### Upregulation of SLC39A6 in precancerous lesions and ESCC tissues and cell lines

**Fig. 1:**
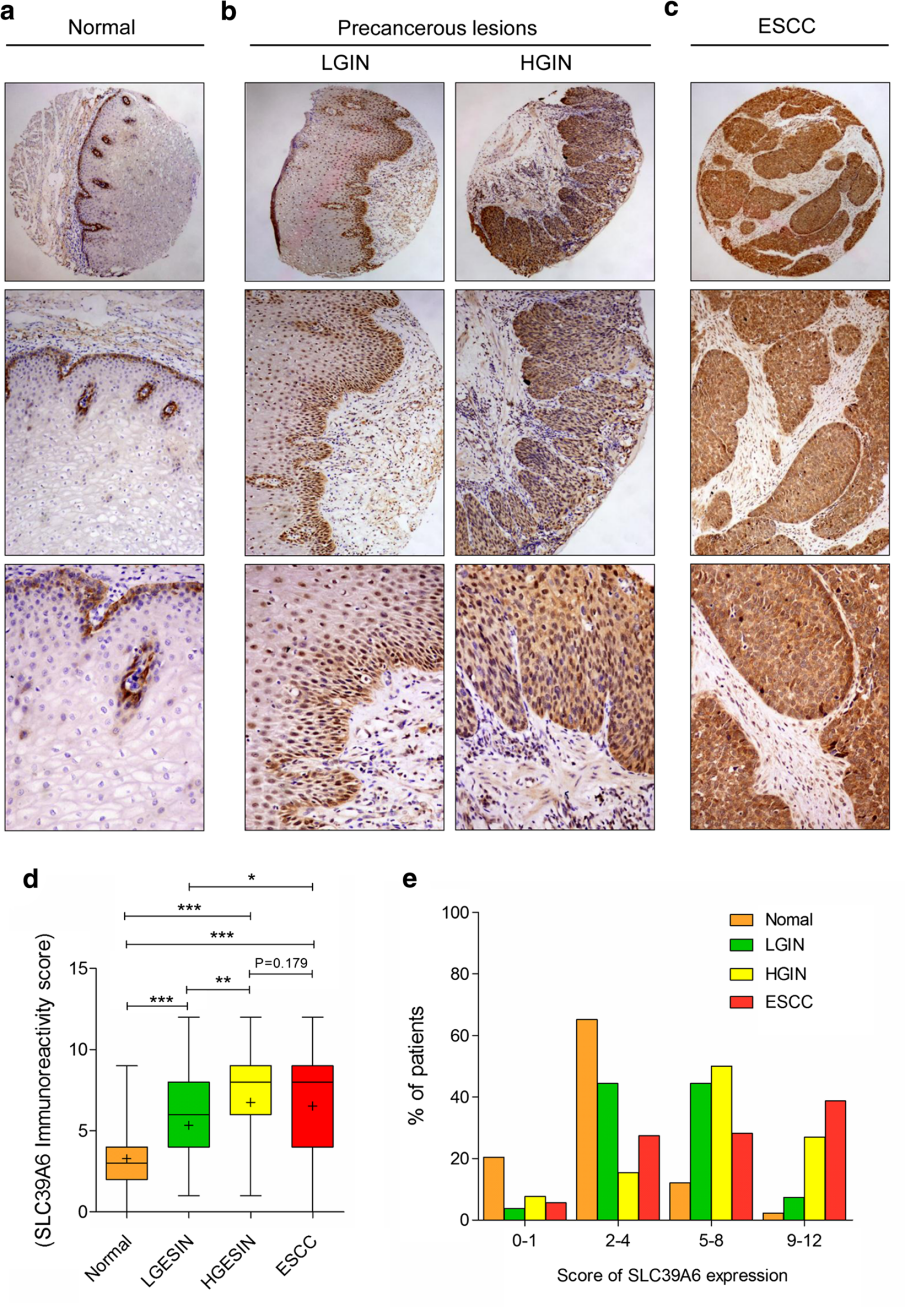
Immunohistochemical analysis of SLC39A6 in non-tumor esophageal, precancerous lesions, and ESCC tissues from Chinese Han ethnic. Representative SLC39A6 immunostaining in **a** non-tumor esophageal (*top panel* magnification ×40; *middle panel* magnification ×100; *bottom panels* magnification ×200), precancerous lesions. **b** LGIN and HGIN (*top panel* magnification ×40; *middle panel* magnification ×100; *bottom panels* magnification ×200), and **c** ESCC tissues (*top panel* magnification ×40; *middle panel* magnification ×100; *bottom panels* magnification ×200). **d** SLC39A6 immunoreactivity was scored in human normal esophageal squamous epithelium, precancerous lesions, and ESCC tissues. (*p < 0.05; **p < 0.01; ***p < 0.001). **e** The four level score (*0–1*, *2–4*, *5–8*, and *9–12*) distribution of SLC39A6 protein expression in normal, precancerous lesions, and ESCC

During the multi-stage development of ESCC in the Chinese Han population, IHC analysis showed that the frequency of SLC39A6 protein overexpression was lowest in normal samples (14.39 %) and increased gradually during the evolution of esophageal carcinogenesis, with 51.85 % (42/81) of LGIN and 76.47 % (39/52) of HGIN, and a slight decrease at 66.90 % (95/142) of ESCC but still showing high SLC39A6 protein expression. In NEE, SLC39A6 labeling was weak and predominantly expressed in the cytoplasm of basal cells and suprabasal layer cells (Fig. 1a); however, SLC39A6 was highly expressed in HGIN and ESCC cells with the signal strongest in nuclei/cytoplasm (Fig. 1b, c). Boxplot showed that the trend of SLC39A6 immunoreactivity score increased in a stepwise manner from normal, LGIN, and ESCC, and peaked in HGIN using the t tests (Fig. 1d). In addition, the four level score (0–1, 2–4, 5–8, and 9–12) distribution of SLC39A6 protein expression in normal, precancerous lesions, and ESCC was significantly distinct (Fig. 1e). Furthermore, as shown in Table 2, in-depth analysis using the Chi square test revealed that the overexpression rates of SLC39A6 protein increased significantly in LGIN, HGIN, and ESCC compared with normal tissues (P < 0.001). Significant differences were also observed between LGIN and HGIN (P = 0.004), as well as between LGIN and ESCC (P = 0.037), whereas no significant differences were found between LGIN and ESCC (P > 0.05, Table 2). However, using the McNemar test, we did not found the SLC39A6 expression significantly differs between ESCC and their corresponding HGIN tissues (P = 0.076); similarly, the SLC39A6 expression also does not significantly differ between ESCC and their corresponding LGIN tissues (P = 0.229; Additional file 3: Table S2).

**Table 2:** SLC39A6 protein expression during cancer progression by IHC analysis in Chinese Han population

Cancer progression	Immunostaining		P value
	Low	High	
Normal esophageal epithelium^①^	113 (85.61)	19 (14.39)	①:②P < 0.001; ①:③P < 0.001
Low grade intraepithelial neoplasia^②^	39 (48.15)	42 (51.85)	②:③P = 0.004; ②:④P = 0.037,
High grade intraepithelial neoplasia^③^	12 (23.08)	40 (76.92)	③:④P = 0.179
ESCC^④^	47 (33.10)	95 (66.90)	①:④P < 0.001

**Fig. 2:**
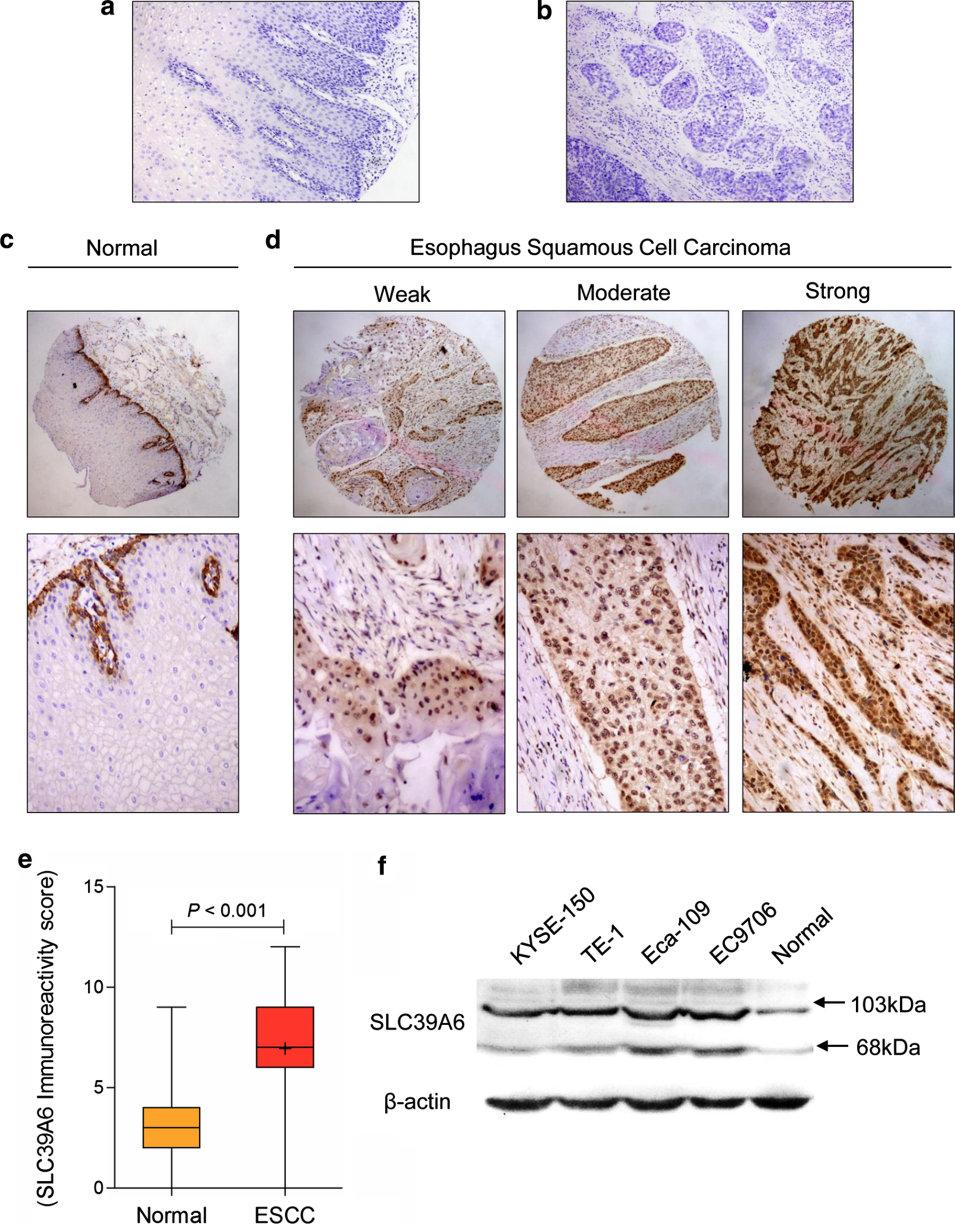
Representative immunohistochemical staining of SLC39A6 in Kazakh ethnic. The negative controls for the normal tissues (**a**) and esophageal cancer (**b**) specimens (magnification ×100). Representative SLC39A6 immunostaining in non-tumor esophageal (**c**) and esophagus squamous cell carcinoma (**d**) tissues with weak, moderate, or strong expression (*top panel* magnification ×40; *middle panel* magnification ×100; *bottom panels* magnification ×200). **e** *Boxplot* shows that SLC39A6 expression levels in ESCC are significantly higher than that in normal esophageal squamous epithelium from Kazakh population (p < 0.001). **f** Expression levels of SLC39A6 proteins were determined in esophageal carcinoma cell lines (Eca109, EC9706, TE-1, and KYSE-150) and a normal esophageal epithelium cell line (HEEC) using western blotting

SLC39A6 protein expression was then externally validated in the cohort of patients from the Kazakh ethnic group (Table 3). As shown in Fig. 2, the negative controls for the normal tissues (Fig. 2a) and esophageal cancer (Fig. 2b) specimens were negative. Consistent with the results of IHC analysis of SLC39A6 protein alteration in Han ethnic, SLC39A6 expression was upregulated in 68/86 (79.07 %) of ESCC specimens, and only in 14/41 (34.15 %) in normal samples (Fig. 2c, d). The expression levels of SLC39A6 protein in ESCC tissues were significantly higher than those in the corresponding normal tissues in the Kazakh population (*P* < 0.001, Fig. 2e). These findings are in accordance with the results from the Han population. The results indicate no difference in terms of the staining pattern and frequency of SLC39A6 protein expression between different racial tissues types collected from the Chinese Han and Kazakh populations, as well as those residing in western China.

**Table 3:** Dysregulation frequency of SLC39A6 protein expression in validation Cohorts

Marker	Han ethnic (n = 142)		Kazakh ethnic (n = 86)		P value
	No.	%	No.	%	
SLC39A6					0.049
Nondysregulated	47	33.1	18	20.93	
Dysregulated	95	66.9	68	79.07

We then examined the expression of SLC39A6 protein in NEE cell line (HEEC) and ESCC cells by Western blotting. Interestingly, we carefully checked the X-ray film and found that all of the lanes of the SLC39A6 protein from the cell lines exhibit two bands, particularly at 68 and 103 kDa. Full-length SLC39A6, as the pro-protein, is predicted to be 85 kDa and produces a 103 kDa band, but this band would be reduced to 68 kDa when SLC39A6 is activated by N-terminal cleavage which triggers SLC39A6 plasma membrane location and zinc influx [20]. Similarly, as shown in Fig. 2F, all esophageal cancer cell lines expressed high levels of modified SLC39A6 protein (68 kDa) compared with NEE cell. Aberrant SLC39A6 expression in both ESCC tissues and ESCC cells suggests that increased SLC39A6 expression might be associated with tumor progression.

### Correlation of SLC39A6 expression with clinicopathologic characteristics

We further determined the association between SLC39A6 expression and clinicopathological parameters of ESCC from the two different populations (Table 4). The expression of SLC39A6 was positively correlated with the histological grade in the Chinese Han and Kazakh populations (P_Han_ = 0.018, P_Kazakh_ = 0.003, respectively). The staining pattern and frequency of SLC39A6 expression were weakly related to lymph node metastasis (p = 0.050), but the effects did not reach significant statistic level. By contract, no significant correlations with age, gender, primary tumor sites, and TNM stage were observed in the Chinese Han ethnic group. When the clinical stage of ESCC in patients was classified into stage I to IV according to the TNM classification, stage I and II tumors showed significantly higher percentages of SLC39A6-positive cells compared with stages III and IV tumors in the Kazakh population (P = 0.026). No statistically significant relationship was observed between SLC39A6 expression and other clinicopathologic characteristics (P > 0.05).

**Table 4:** The correlations between SLC39A6 protein expression and clinicopathologic characteristics in Kazakh and Han ethnic

Variables	SLC39A6 expression in Han ethnic				SLC39A6 expression in Kazakh ethnic			
	Total cases	Low n (%)	High n (%)	P value	Total cases	Low n (%)	High n (%)	P value
Gender				0.863				0.621
Male	107	35 (32.71)	72 (67.29)		53	12 (22.64)	41 (77.36)	
Female	35	12 (34.29)	23 (65.71)		33	6 (18.18)	27 (81.82)	
Age (years)				0.254				0.113
≤60	57	22 (38.60)	35 (61.40)		53	14 (26.42)	39 (73.58)	
>60	85	25 (29.41)	60 (70.59)		33	4 (12.12)	29 (87.88)	
Differentiation^a^				0.018				0.003
Well	39	20 (51.28)	19 (48.72)		21	10 (47.62)	11 (52.38)	
Moderate	74	19 (25.68)	55 (74.32)		53	6 (11.32)	47 (88.68)	
Poor	29	8 (27.59)	21 (72.41)		12	2 (16.67)	10 (83.33)	
Lymph node metastasis				0.050				0.690
No	93	36 (38.71)	57 (61.29)		37	7 (18.92)	30 (81.08)	
Yes	49	11 (22.45)	38 (77.55)		49	11 (22.45)	38 (77.55)	
TNM stage				0.276				0.026
I + II	94	34 (36.17)	60 (63.83)		53	7 (13.21)	46 (86.79)	
III + IV	48	13 (27.01)	35 (72.99)		33	11 (33.33)	22 (66.67)	

P < 0.05 indicates a significant association among the variables

^a^Well-differentiation vs. moderate differentiation + poor differentiation

### Sensitivity and specificity values of SLC39A6 in ESCC

**Fig. 3:**
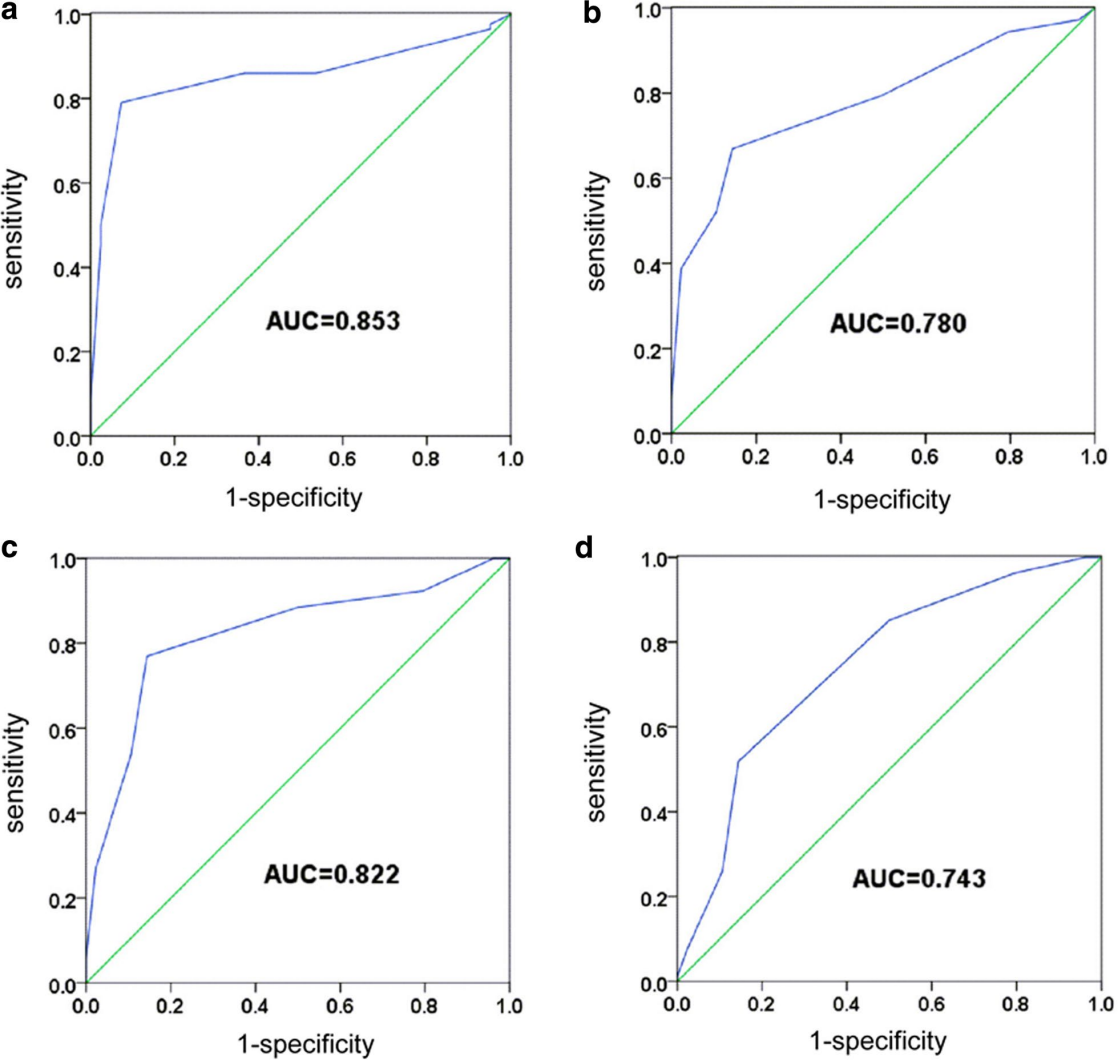
Receiver operating characteristic curve analysis for SLC39A6 immunohistochemical scores for detecting precancerous lesions and ESCC tissues. **a** Kazakh ethnic ESCC tissues. **b** Han ESCC tissues. **c** HGIN tissues. **d** LGIN tissues. The AUC in Kazakh ESCC, Han ethnic ESCC, HGIN, and LGIN is 0.853, 0.78, 0.822, and 0.743, respectively

Using the distant normal mucosa as the control, the receiver operating characteristic (ROC) curves for various types of tissues clearly illustrate the point on the curve closest to (0.0, 1.0), which maximizes both sensitivity and specificity for ESCC, HGIN, and LGIN. The score with the most areas under the ROC curve (AUC) and having both maximum “sensitivity” and “1-specificity” was selected as the cut-off score. We found that the immunohistochemical cut-off scores of SLC39A6 easily distinguished the ESCC, HGIN, and LGIN tissues from the normal esophageal tissues, all of which demonstrated high sensitivity and specificity values according to the area under ROC curve (all >0.7, Fig. 3; Additional file 4: Table S3). These results support the notion that SLC39A6 may be a potential diagnostic biomarker for ESCC and ESIN.

### SLC39A6 overexpression predicts poor prognosis in ESCC

**Fig. 4:**
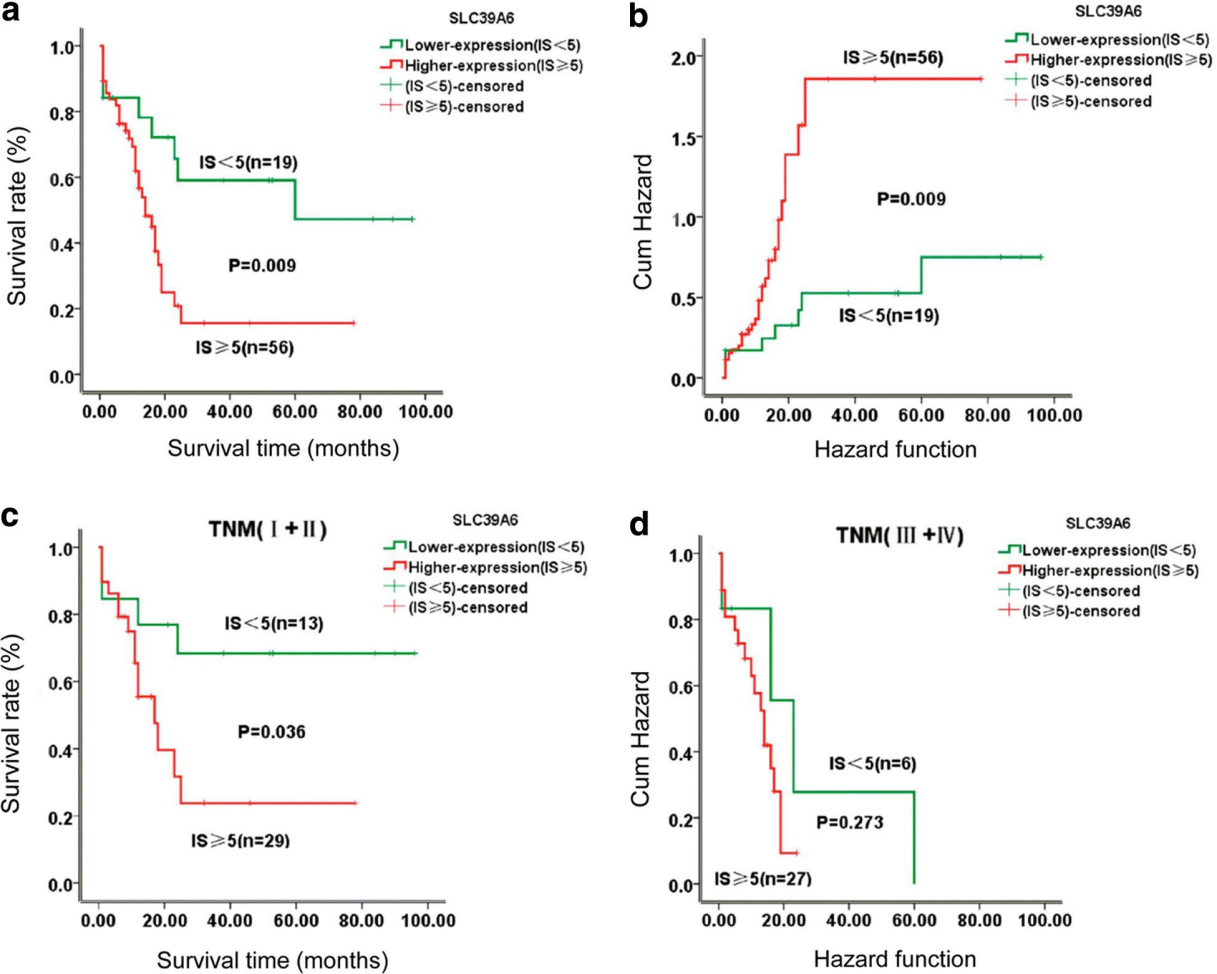
Kaplan–Meier survival curves for patients with overexpressed SLC39A6 and those with low SLC39A6 levels. **a** ESCC patients with overexpressed SLC39A6 (IS ≥ 5) experienced a significantly shorter survival period after surgery than those with low SLC39A6 levels (IS < 5) (p < 0.01). **b** Patients with SLC39A6 overexpression had a greater risk of death than those with lower SLC39A6 levels (p < 0.01). **c**, **d** Patients with high SLC39A6 expression shows the worse overall survival at stage I + II than those with low-expression (p < 0.05)

The association between SLC39A6 protein expression and overall survival (OS) of ESCC was estimated using log-rank test and multivariable Cox proportional hazard regression analysis. Of the 142 ESCC patients examined from the Han ethnic, clinical follow-up information was available for 75 patients. The median survival time of patients with lower SLC39A6 expression was 38.21 months (range 1–96 months), whereas that of patients with SLC39A6 overexpression was only 12.18 months (range 1–78 months). As shown in Fig. 4a, Kaplan–Meier survival analysis showed that ESCC patients with higher expression of SLC39A6 protein had significantly worse prognosis than ESCC patients with low or no expression (log-rank test, χ^2^ = 6.749, P = 0.009). Patients with SLC39A6 overexpression had worse OS and greater risk of death after surgery than those with a weak or negative SLC39A6 expression (P = 0.009, Fig. 4b). Furthermore, when the ESCC patients were stratified according to clinical stage, the survival rates of patients with SLC39A6 overexpression cancer were significantly lower than those of patients with SLC39A6 down-expression in early stage ESCC (P = 0.036, Fig. 4c). By contrast, SLC39A6 was not related to ESCC survival in later stage ESCC (P = 0.273, Fig. 4d).

To identify independent prognostic factors for ESCC survival, univariate and multivariate Cox regression models were used. Univariate Cox proportional hazard regression analysis revealed that lymph node metastasis [hazard ratio (HR) = 2.315, 95 % CI 1.226–4.371, P = 0.010], clinical stage (HR = 2.146, 95 % CI 1.134–4.063, P = 0.019), and SLC39A6 (HR = 2.780, 95 % CI 1.230–6.281, P = 0.014) were significant prognostic predictors for OS of ESCC patients in the entire population (Table 5). Other clinicopathological parameters, including gender, age, and differentiation, were not prognostic factors for OS in our study (Table 5). Furthermore, it appears that SLC39A6 protein was an independent prognostic factor of ESCC (HR = 2.536, 95 % CI 1.079–5.962, P = 0.033) in the multivariate Cox regression models (Table 5). Together, these data indicate that SLC39A6 overexpression was a significant independent prognostic factor for poor prognosis in ESCC and could thus be used as a potential biomarker for prognosis evaluation in patients with ESCC.

**Table 5:** Univariate and multivariate Cox regression analyses of the prognostic variables in ESCC patients

Variables	Univariate analysis				Multivariate analysis			
	HR	95 % CI		P value	HR	95 % CI		P value
SLC39A6 expression	2.780	1.230	6.281	0.014	2.536	1.079	5.962	0.033
Gender (female)	1.400	0.706	2.773	0.335	1.522	0.734	3.157	0.259
Age (>60 years)	0.843	0.443	1.602	0.601	0.914	0.466	1.79	0.793
Differentiation (moderate)	0.798	0.314	2.027	0.636	0.963	0.370	2.509	0.939
Differentiation (poor)	0.886	0.393	1.998	0.770	0.879	0.373	2.072	0.768
Lymph node metastasis (yes)	2.315	1.226	4.371	0.010	2.129	0.611	7.412	0.235
TNM stage (III + IV)	2.146	1.134	4.063	0.019	1.034	0.303	3.523	0.958

Significant difference that 95 % CI of HR was not including

*HR* hazard radio, *CI* confidence interval

### Downregulation of SLC39A6 suppresses ESCC cell growth and induces apoptosis

**Fig. 5:**
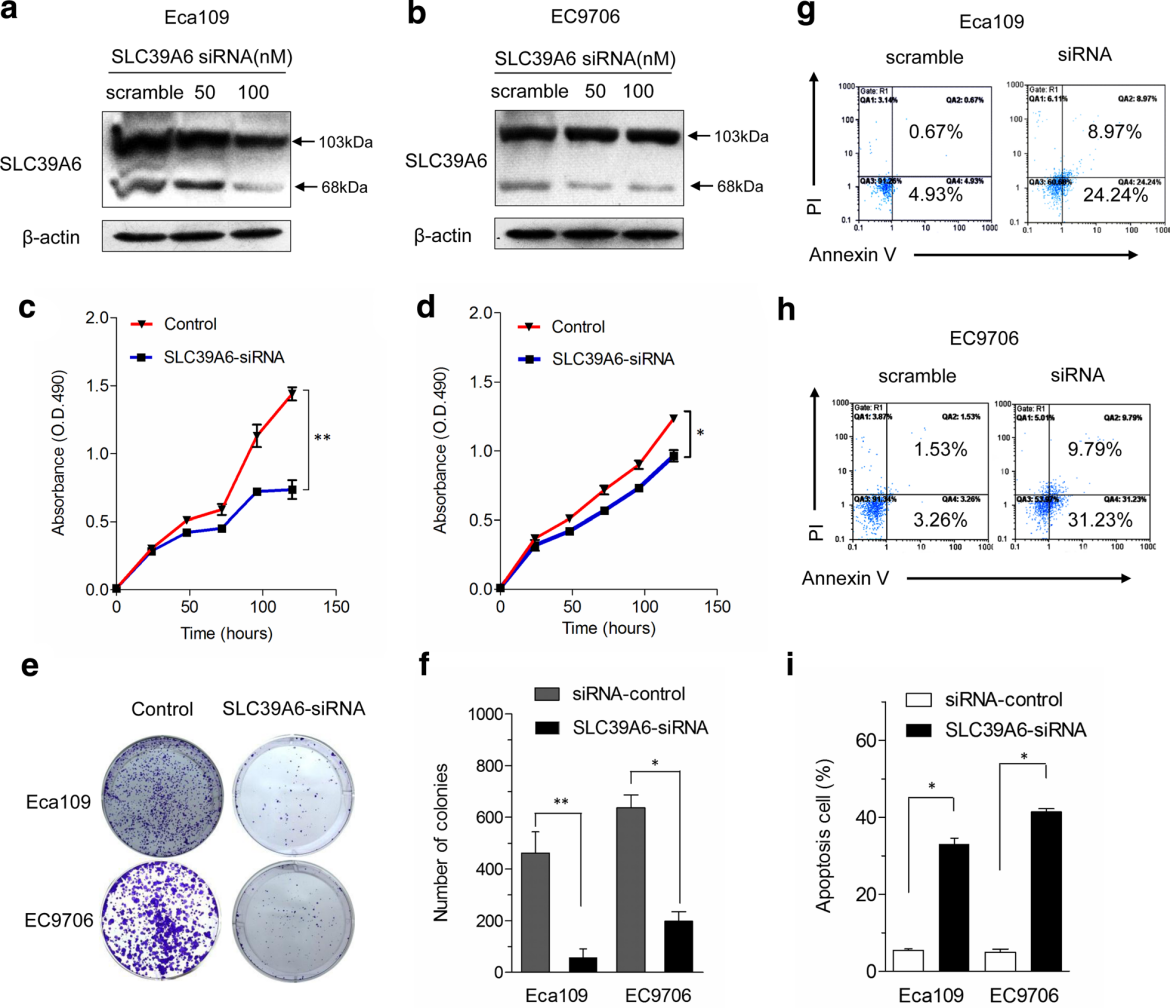
Knockdown of SLC39A6 inhibits cell growth and enhances cell apoptosis in vitro. **a**, **b** Eca-109 and EC9706 cells were transfected with either SLC39A6-siRNA or control siRNA after 72 h, the cells were collected, and total cellular protein was used for western blotting analysis with anti-SLC39A6 antibody as described. β-actin served as an internal control. **c**, **d** Silencing endogenous SLC39A6 inhibits cell growth as determined by MTT assays. **e** Silencing endogenous SLC39A6 inhibits cell growth as determined by colony formation assays. **f** The histograms indicate that the number of colonies formed by cells treated with SLC39A6 siRNA was far fewer than that of control siRNA-treated cells (*p < 0.05; **p < 0.01). **g**, **h** Cell apoptosis was detected by Annexin-V/propidium iodide combined labeling flow cytometry in Eca-109 and EC9706 cells upon inhibition of SLC39A6 protein. Flow cytometry analysis shows a large increase in the percentage of cells programmed for apoptosis in Eca-109-siRNA and EC9706-siRNA cells comparing to the corresponding negative controls. **i** Statistics of the results in (**g**) and (**h**). *p < 0.05, versus scramble control (Student’s t test). *Error bars* represent mean ± SD from three independent experiments

To ascertain that SLC39A6 is essential for esophageal cancer cell proliferation, we used siRNAs to deplete SLC39A6 protein in ESCC cells. Western blot analysis demonstrated an efficient knockdown of over 60 % of SLC39A6 protein expression after 72 h of transfection of siRNA against SLC39A6 in a dose-dependent manner but not by control siRNA in Eca109 and EC9706 cell lines (Fig. 5a, b). Cell growth was analyzed using MTT assay to determine whether downregulation of SLC39A6 has an inhibitory effect on ESCC cell proliferation. Growth curves demonstrated that growth of SLC39A6 siRNA-mediated cells was significantly inhibited compared with that of the control group (Fig. 5c, d). For the colony formation assay, the colonies from SLC39A6 siRNA-mediated cells were much smaller than those from the control cells (Fig. 5e), and the number of colonies decreased by an average of four- to eightfold in ECA109 and EC9706 cells compared with that in the control cells (P < 0.05, n = 3, Fig. 5f).

We performed flow cytometric analysis using Annexin V + (early apoptosis marker) and Annexin V +/PI + (late apoptosis) cells to investigate the effect of SLC39A6 knockdown on apoptosis. As shown in Fig. 5g–i, the results show a higher frequency of cells programmed for both early and late phases of apoptosis in SLC39A6 knockdown cells compared with that in vector controls. We observed an average increase of 5.39- and 9.57-fold in the early apoptosis rate in Eca-109-SLC39A6-siRNA and EC9706-SLC39A6-siRNA cells compared with that in negative control cells. The late apoptosis rate for these cells increased by 13.38- and 6.39-fold respectively, indicating that downregulation of SLC39A6 promoted the apoptosis of ESCC cells.

### SLC39A6 induce the EMT phenotype and increases ESCC cells invasiveness

**Fig. 6:**
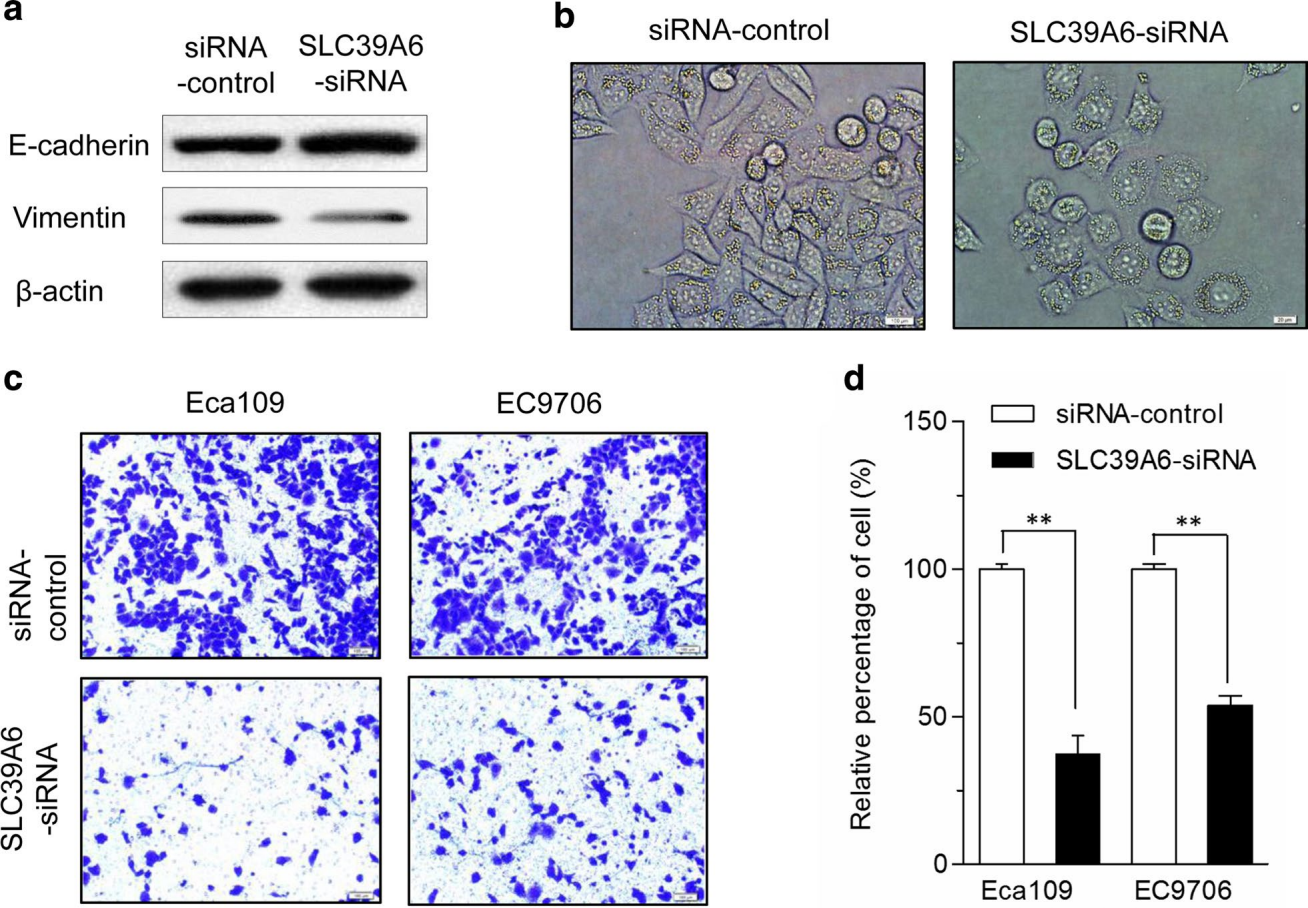
SLC39A6 promotes the EMT phenotype and inhibition of SLC39A6 expression diminished invasion capacity of Eca109 and EC9706 cells. **a** SLC39A6-siRNA was transfected into Eca109 and EC9706 cells for 72 h and cells were harvested for an immunoblot analysis of vimentin and E-cadherin. Silencing SLC39A6 was accompanied by the increased expression of E-cadherin and loss of vimentin, which are all hallmarks of EMT markers. Knockdown of SLC39A6 results in morphological changes in Eca109 cell. **b** Photographs were taken using a Nikon microscope (phase contrast). Original magnification ×200. **c** Cell invasion assay was performed using Matrigel-coated transwell plates for Eca109 and EC9706 cells. Knockdown of SLC39A6 significantly reduced cell invasiveness in the two esophageal cancer cell lines compared with that in SLC39A6-siRNA controls. **d** The relative percentage of cells passing through a Matrigel filter (**p < 0.01). All experiments were performed at least three times with consistent and repeatable results

Cancer metastasis is associated with EMT. To investigate whether SLC39A6 regulates EMT in esophageal cancer cells, the epithelial cell marker E-cadherin and mesenchymal marker vimentin were examined in Eca109 cell transfected with SLC39A6-siRNA and controls using the western blot. The epithelial marker E-cadherin was significantly upregulated, whereas the mesenchymal markers vimentin was significantly reduced in Eca109 cells with knockdown of SLC39A6 compared with the controls siRNA groups (Fig. 6a). In addition, cell morphology examination indicated that knockdown of SLC39A6 resulted in morphological changes in Eca109 cells (Fig. 6b).

In the reciprocal experiments, we examined whether knocking down SLC39A6 would inhibit ESCC cell invasiveness using SLC39A6 siRNA. As shown in Fig. 6c, ESCC cell invasive ability significantly decreased in SLC39A6-siRNA-treated cells compared with the control siRNA groups. The number of cells that invaded the Matrigel membrane was significantly lowered in SLC39A6-siRNA-treated cells compared with that in the control groups (P < 0.01) (Fig. 6d).

## Discussion

A multistage process has been proposed for the evolution of ESCC, in which normal squamous epithelia undergo a series of histological and genetic progression towards noninvasive precursor lesions, and then towards invasive cancer. Although a variety of molecular alterations have been identified over the last two decades, sensitive and specific biomarkers for early diagnosis and accurate indicators for ESCC prognosis are currently unavailable. Therefore, identification of targets for early detection of ESCC is important to improve the prognosis of patients with this pernicious disease. The key finding in this report lies in, for the first time, SLC39A6 protein level was both elevated in Chinese Han and Kazakh ethnic patients with ESCC, and this finding was also observed in precursor lesions tissues. In addition, in vivo assays indicated the biological role of SLC39A6 in promoting anti-apoptosis and invasiveness by inducing the EMT phenotype of ESCC, further cementing that targeting of SLC39A6 might be potential therapeutic strategy for blocking ESCC.

SLC39A6, a member of a new subfamily of zinc transporters, is involved in maintaining the intracellular homeostasis of zinc, an ion that is essential in the control of gene transcription, differentiation, development and growth, suggesting that its altered distribution might promote tumorigenesis [12, 21]. Accumulating evidence demonstrated that SLC39A6 may be linked to carcinogenic processes. Previous studies have shown that SLC39A6 is overexpressed in breast, prostate, pancreatic, cervical, and liver cancers [15–18, 22]. In ESCC, a previous study showed that SLC39A6 overexpression in Chinese Han ethnic ESCC patients is a susceptibility gene for ESCC in a Chinese Han population [10]. However, whether SLC39A6 expression is increased in precursor lesions of ESCC and other ESCC patients of different ethnicities needs further exploration. Herein, we firstly demonstrated that SLC39A6 protein expression increased progressively from NEE to LGIN to ESCC, and peaked in HGIN in the Han ethnic cohort. We further analyzed the relationship between SLC39A6 protein expression and outcome of patients with ESCC. Our results show that patients with higher SLC39A6 expression in ESCC tissues had a worse prognosis than those with lower SLC39A6 expression. Cox regression analysis confirmed that SLC39A6 expression was an independent prognostic factor for poor OS in ESCC. All these results indicate that SLC39A6 overexpression could increase immediately when the esophageal squamous epithelium changed, as an “early” and “intermediate” event in the ESCC malignant progression, indicating that SLC39A6 could serve as early detector of high-risk subjects and prognostic biomarker. Similarly, SLC39A6 protein was significantly overexpressed in Kazakh ethnic ESCC compared with that in NEE. Although the two ethnic groups had different customs, cultural backgrounds, and food habits, in our study, overexpression of SLC39A6 was observed in both Han and Kazakhs ESCC tissues. This finding was in agreement with the findings in a Chinese Han population by Wu et al. [10], which supported the hypothesis that SLC39A6 may function as an oncogene in ESCC patients from the two ethnic groups, Han and Kazakhs. To our knowledge, this study is the first report on the SLC39A6 protein in the precursor lesions of ESCC and Kazakh populations.

Interestingly, in the present study, we also made an observation that SLC39A6 is expressed as a pro-protein (103 kDa) in inactive and active forms (68 kDa) in all of the esophageal cell lines. This result is consistent with that observed by Taylor et al. [20] in breast cancer cell lines; Taylor KM et al. [20] found three SLC39A6 protein bands, particularly those at 103, 68, and 35 kDa; these bands are obtained because of the N-terminal proteolytic cleavage of SLC39A6 by using three anti-SLC39A6 antibodies. SLC39A6 is modified via N-terminal cleavage before this protein relocates to the plasma membrane in breast cancer. However, whether SLC39A6 is activated via N-terminal cleavage in esophageal cancer should be clarified in future studies.

The role of zinc in cell growth, division, and differentiation, as well as basal homeostasis, is of key importance [13]. Loss of terminal differentiation is one of the characteristics of ESCC cells, even in the well-differentiated cases, and the differentiation methods of cancer cells were quite different from those of normal non-keratinised squamous epithelium. We have, for the first time, independently observed that high SLC39A6 expression in Han and Kazakh patients with ESCC was associated with poor differentiation. Changes in cellular zinc concentrations in various types of cancers [23] are likely the result of alterations in the expression of zinc transporters [24–26]. Thus, we speculate that increased SLC39A6 promotes the loss of terminal differentiation in normal non-keratinized squamous epithelium. We also observed that high SLC39A6 expression in Kazakh patients was associated with TNM stage, but differ from those in Han. This discrepancy may be due to several factors, of which sample size may be one as Kazakh cohort had a smaller sample size (n = 82) than the Han cohort (n = 142). The other possible factors resulting in the discrepancy may be population heterogeneity and genetic backgrounds of different ethnicities, which need to further clarified in studies using uniform ethnic groups with larger sample sizes.

SLC39A6 is involved in maintaining the intracellular homeostasis of zinc, an ion that is essential for cell proliferation and tumor growth [12, 21]. The cell proliferating effects of growth factors are accompanied with an increase in the concentrations of labile zinc [13]. Here, indirectly consistent with these previous reports, we found that knockdown of SLC39A6 expression significantly reduced proliferation and promoted ESCC cell apoptosis. Our findings were in consistent with those of Betty et al., who found that dietary zinc depletion is effective in retarding tumor growth even with well-established tumors [27]. However, the mechanism by which SLC39A6 induces the proliferation of ESCC remains unclear. Indirect evidence from Chen et al., confirmed that more zinc-deficient cells remain in the S phase and do not undergo mitosis compared with zinc-sufficient cells, with cell proliferation being attenuated based on cell cycle studies [28]. Our studies indicate that the targeting of SLC39A6 might be potential therapeutic strategy for blocking ESCC proliferation by restriction specific minerals, such as zinc, via knockdown of SLC39A6.

Invasion and metastasis are two of the most important hallmarks of cancer [23]. SLC39A6 has been reported to be a downstream target of signal transducer and activator of transcription 3 (STAT3). It plays a critical part in gastrula organizer cells undergoing EMT by affecting Snail activity [29]. SLC39A6 was linked to MET in breast cancer through SLC39A6-induced zinc influx when N-terminal cleavage inactivates GSK-3β; as a result, unphosphorylated Snail in the nucleus downregulates the adherence genes, such as E-cadherin. Thus, cell rounding and detachment occur [20]. Considering the potential mechanism of the SLC39A6 in ESCC, we performed in vitro functional analyses in the current study and showed that the suppression of the SLC39A6 expression modulated the malignant phenotype of ESCC cells, abrogated cell invasion, and induced an EMT phenotype in ESCC cell lines. The expression of E-cadherin, loss of vimentin, and morphological changes in ESCC cells were also enhanced in vitro. EMT is a consistently observed phenomenon that is a vital aspect of embryogenesis and cancer progression [30]. During EMT, cancer cells lose their adhesion and begin the process of metastasis [31]. SLC39A6 was shown to be a mediator downstream from the STAT3 and Snail, cooperating with Snail in the repression of epithelial marker E-cadherin gene transcription [29]. Given that the induction of EMT by ectopic expression of either the Twist or Snail transcription factors generated stem cell-like cells [32], we proposed that SLC39A6 might regulate the stemness of esophageal cancer and promote the invasion of ESCC cells by affecting the expression of STAT3 and Snail, which needs further exploration.

However, it must be pointed out that we did not found SLC39A6 expression significantly differs between ESCC and their corresponding HGIN tissues; similarly, the SLC39A6 expression does not significantly differ between ESCC and their corresponding LGIN tissues (Additional file 3: Table S2), which was in conflict with the results provided in Table 2 between LGIN and ESCC (P = 0.037). There could be multiple reasons contributed to the inconsistency in results. However, as all we known, it’s impossible for us to find the patients with only precancerous lesions. Thus, we selected the adjacent esophageal cancer tissues from the patients with ESCC diagnosed as HGIN or LGIN tissues to compare the SLC39A6 expression among the different characteristics of the tissues, enhance the diagnostic value of the early detection of ESCC, and to clarify the function of SLC39A6 as a biomarker of the early detection of ESCC; nevertheless, this phenomenon is also the limitation of our research. The current results reveal the difference among HGIN, LGIN, and ESCC. To a certain degree, these results indicate that the SLC39A6 expression can be used as a marker to detect ESCC in early stages. However, we also acknowledge that the sampling selection in the present study has many limitations.

## Conclusions

In summary, our study reveals that SLC39A6 was upregulated in the onset of ESCC. Thus, SLC39A6 may be a potential biomarker for early detection of high-risk subjects and early diagnosis of ESCC and an independent prognosis factor of ESCC. Moreover, knockdown of SLC39A6 expression modulated the malignant phenotype of ESCC cells, resulting in significantly reduced proliferation and invasion with MET phenotype in vitro. Combined, our findings highlight a tumor-promoting role for SLC39A6 in ESCC and targeting SLC39A6 might be a potential therapeutic strategy.

## Additional files

10.1186/s12967-015-0681-z The number of different kinds of the esophageal tissues and the overlapping sample among them.
10.1186/s12967-015-0681-z The clinical-pathological characteristics of 75 esophageal cancer patients with follow-up information.
10.1186/s12967-015-0681-z The comparison of SLC39A6 protein expression between ESCC and their corresponding LGIN and HGIN tissues in Chinese Han population
10.1186/s12967-015-0681-z The high sensitivity, specificity and AUC values of SLC39A6 in ESCC, HGIN, and LGIN
