# Supplementary material for: SLC39A6: a potential target for diagnosis and therapy of esophageal carcinoma
Source: J Transl Med. 2015 Oct 6;13:321. doi: 10.1186/s12967-015-0681-z (PMC4595240; doi:10.1186/s12967-015-0681-z)
Supplement: Supplementary file 1 — 10.1186/s12967-015-0681-z The number of different kinds of the esophageal tissues and the overlapping sample among them. [file 12967_2015_681_MOESM1_ESM.docx]

**Additional file 1: Figure S1. The number of** **different kinds of the esophageal tissues and** **the** **overlapping sample among them**

**
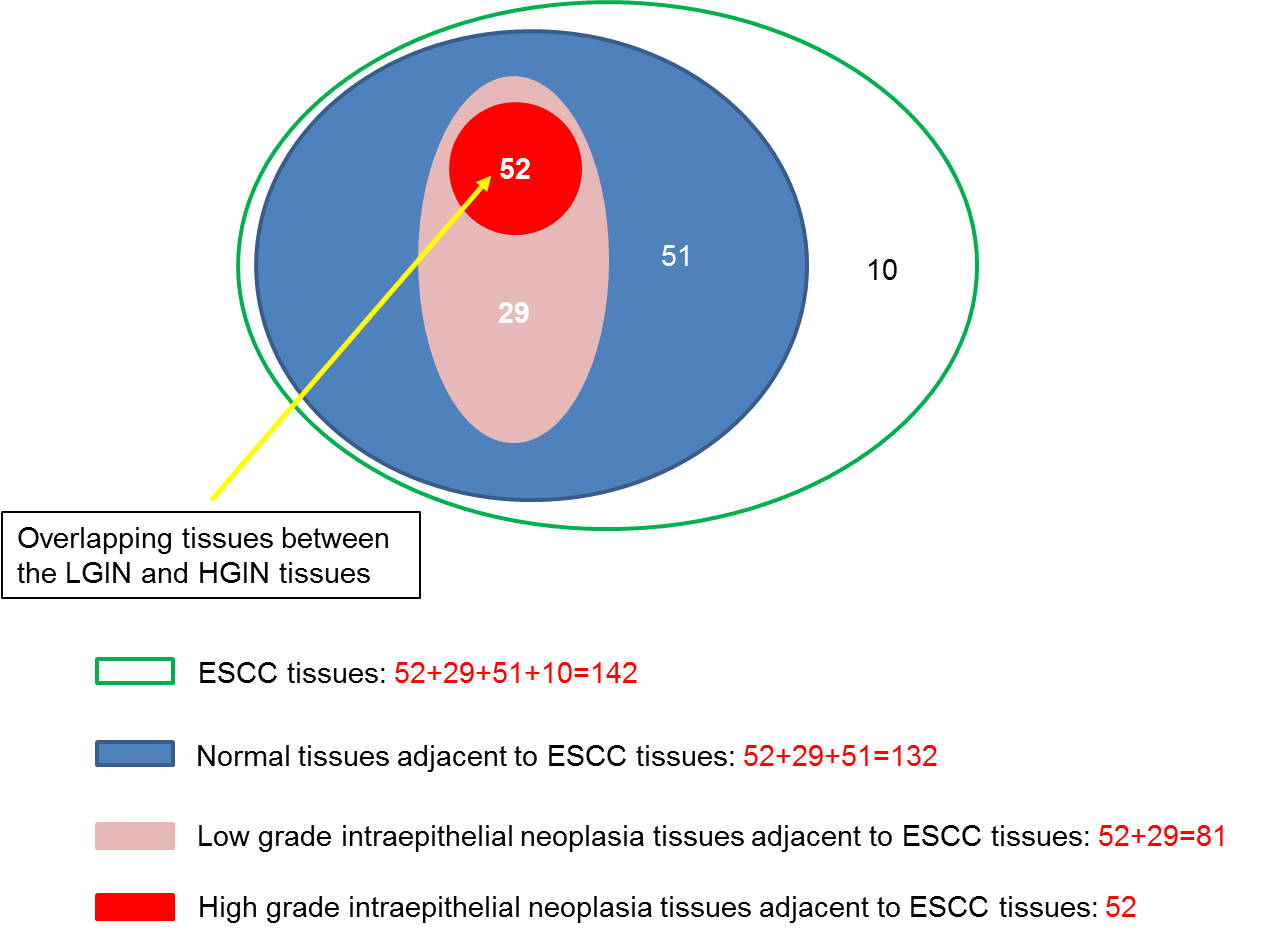
**
