# Supplementary material for: SLC39A6: a potential target for diagnosis and therapy of esophageal carcinoma
Source: J Transl Med. 2015 Oct 6;13:321. doi: 10.1186/s12967-015-0681-z (PMC4595240; doi:10.1186/s12967-015-0681-z)
Supplement: Supplementary file 2 — 10.1186/s12967-015-0681-z The clinical-pathological characteristics of 75 esophageal cancer patients with follow-up information. [file 12967_2015_681_MOESM2_ESM.docx]

**Additional file 2: Table S1.** **The clinical-pathological characteristics of 75 esophageal cancer** **patients with follow-up information**

| **Characteristics** | **No.** | **%** |
| --- | --- | --- |
| **Age** |  |  |
| **median** | 66 |  |
| **range** | 43-81 |  |
| **Sex** |  |  |
| **Male** | 54 | 72 |
| **Female** | 21 | 28 |
| **Didderentiation** |  |  |
| **Well** | 18 | 24 |
| **Moderate** | 42 | 56 |
| **Poor** | 15 | 20 |
| **Lymph node metasis** |  |  |
| **No** | 40 | 53.33 |
| **Yes** | 35 | 46.67 |
| **TNM** |  |  |
| **I+II** | 42 | 56 |
| **III+IV** | 33 | 44 |
| **Time** |  |  |
| **median** | 12 |  |
| **range** | 1-96 |  |
| **Status** |  |  |
| **death** | 40 | 53.33 |
| **survival** | 35 | 46.67 |
| **SLC39A6 expression** |  |  |
| **low** | 19 | 25.33 |
| **high** | 56 | 75.67 |
