# Supplementary material for: SLC39A6: a potential target for diagnosis and therapy of esophageal carcinoma
Source: J Transl Med. 2015 Oct 6;13:321. doi: 10.1186/s12967-015-0681-z (PMC4595240; doi:10.1186/s12967-015-0681-z)
Supplement: Supplementary file 3 — 10.1186/s12967-015-0681-z The comparison of SLC39A6 protein expression between ESCC and their corresponding LGIN and HGIN tissues in Chinese Han population [file 12967_2015_681_MOESM3_ESM.docx]

**Additional file 3:** **Table S2. The comparison of SLC39A6 protein expression between ESCC and their corresponding LGIN and HGIN tissues in Chinese Han population**

| **LGIN** | **ESCC** | | **Total** | **P value** |  | **HGIN** | **ESCC** | | **Total** | **P value** |
| --- | --- | --- | --- | --- | --- | --- | --- | --- | --- | --- |
|  | **Low** | **High** |  |  |  |  | **Low** | **High** |  |  |
| Low | 18 | 21 | 39 | 0.229 |  | Low | 4 | 8 | 12 | 0.076 |
| High | 13 | 29 | 42 |  |  | High | 18 | 22 | 40 |  |
| Total | 31 | 50 | 81 |  |  | Total | 22 | 30 | 52 |  |
