# Supplementary material for: SLC39A6: a potential target for diagnosis and therapy of esophageal carcinoma
Source: J Transl Med. 2015 Oct 6;13:321. doi: 10.1186/s12967-015-0681-z (PMC4595240; doi:10.1186/s12967-015-0681-z)
Supplement: Supplementary file 4 — 10.1186/s12967-015-0681-z The high sensitivity, specificity and AUC values of SLC39A6 in ESCC, HGIN, and LGIN [file 12967_2015_681_MOESM4_ESM.docx]

**Additional file 4: Table S3. The high sensitivity, specificity and AUC values of SLC39A6 in ESCC, HGIN, and L****GIN**

| **Comparison** | **AUC** | **Sensitivity (%)** | **Specifity (%)** | **Cut off value^a^** |
| --- | --- | --- | --- | --- |
| **ESCC versus Normal** |  |  |  |  |
| **Han ethnic** | 0.780 | 77.90 | 85.60 | 5 |
| **Kazakh ethnic** | 0.853 | 79.06 | 92.68 | 5 |
| **HGIN versus Normal** | 0.822 | 76.90 | 85.60 | 5 |
| **LGIN versus Normal** | 0.743 | 51.90 | 85.60 | 5 |

**^a^** Cut off level was set to provide optimal sensitivity and specificity.
